# Treating Patients Like Athletes: Sports Science Applied to Parkinson's Disease

**DOI:** 10.3389/fneur.2020.00228

**Published:** 2020-04-02

**Authors:** Raquel Bouça-Machado, Massimo Venturelli, Michele Tinazzi, Federico Schena, Joaquim J. Ferreira

**Affiliations:** ^1^Instituto de Medicina Molecular, Faculdade de Medicina, Universidade de Lisboa, Lisbon, Portugal; ^2^CNS-Campus Neurológico Sénior, Torres Vedras, Portugal; ^3^Department of Neurosciences, Biomedicine and Movement Sciences, University of Verona, Verona, Italy; ^4^Laboratory of Clinical Pharmacology and Therapeutics, Faculdade de Medicina, Universidade de Lisboa, Lisbon, Portugal

**Keywords:** Parkinson's disease, exercise, principles of training, sport science, disease management, integrated care

## Abstract

The evidence demonstrating the benefits of exercise is indisputable for healthy subjects, and more recently, it is growing for Parkinson's disease (PD) patients. Due to its easy access, low cost, social facilitation and, above all, the symptomatic effect, clinical exercise may have a profound impact on PD management. Especially considering that in recent decades there have been no major advances from the pharmacological point of view. Despite this, clinical exercise use it stills limited by the existent flaws in the available evidence supporting its use and guiding its prescription as a PD therapeutic intervention. We believe that a approach from the most relevant scientific and clinical fields is crucial to establish the use of clinical exercise in PD patients' routine care. Therefore, in this viewpoint, we aim to highlight the importance of clinical exercise as a therapeutic intervention in PD, and particularly of the benefits of applying sports science principles to potentiate the use of clinical exercise as a therapeutic intervention in PD management.

Clinical exercise is a subtype of physical activity (PA), defined as the implementation of the sports science knowledge involving physiologic, metabolic, and structural responses and prescribing principles to short- and long-term PA with clinical relevance to the management of health conditions (1–3).

Parkinson's disease (PD) is currently the second most common neurodegenerative disease and it is expected that its prevalence doubles over the next 20 years (4). Although several pharmacological and surgical therapies proved to be effective for symptomatic control, there is no effective disease modifying treatment yet, and the available symptomatic therapies have several flaws (5–7). Clinical exercise has been recommended as a complement to pharmacological interventions for the management of PD symptoms unresponsive to drug therapy, being presently also recognized as one of the most promising therapeutic interventions with a disease-modifying effect (8–10).

Nonetheless, clinical exercise it still often not recognized as part of PD armamentarium with only 47% complying with physical activity standard recommendations for adults (11).

With this viewpoint, we seek to highlight the importance of exercise as a therapeutic intervention in PD and particularly of the benefits of applying sports science principles to potentiate the use of exercise as a therapeutic intervention in PD management.

## Current Views on the Benefits of Exercise in PD

Presently, there is a strong consensus that increasing the PA levels of PD patients improves symptom management, physiological and structural brain function (12).

Recent studies have also shown different benefits associated with different types of exercise. In concrete: endurance training has been associated with benefits on tremor, bradykinesia, physical fitness, balance, gait speed, motor function, quality of life, and falls risk (10, 13–16); resistance training with benefits improving muscle strength, balance, functional mobility, and quality of life; and balance training seems to improve patients' mobility and postural instability (Table 1) (10, 12, 17, 18). In addition, a 2017 case-control reported an improvement of motor functions and whole-body metabolism in early/mid-stage PD patients, after 3-months of combined endurance and strength training (19).

**Table 1:** Positive results of the different types of exercise.

**Exercise type**	**Benefits**
Endurance training	-Tremor - Bradykinesia - Physical fitness -Balance - Gait speed - Motor function - Quality of life - Fall risk
Resistance training	-Muscle strength -Balance -Functional mobility -Quality of life
Balance training	-Patients' mobility -Cadence and gait velocity - Postural instability

Although structured exercise programs are the most efficacious way to comply with the recommended levels of PA, some preceding steps may be needed, because of the sedentary lifestyle of most PD patients (6, 20). Some patients may need to start by simply being more active throughout the day, promoting different possible forms of PA, such as parking away from the site and walking, doing housework, climbing stairs, or gardening (20–22). As patients improve their physical capacity, further benefits may be acquired with structured exercise programs of moderate or high intensity (21, 22).

According to recent published studies on exercise interventions in PD, the heterogeneity of interventions tested, the lack of standardize outcomes and the low quality of reporting of the studies, affects the possibility of summarizing research findings, limiting the possibility of establishing the most suitable type, frequency, duration and intensity of clinical exercise for each stage of PD (13, 23).

## How Can Sport Science Principles Potentiate Clinical Exercise Interventions in PD?

For a training session to be considered successful, supercompensation should be achieved. This is the leading concept of exercise training and is usually defined as an adaptive response following a controlled application of stress on the body and its subsequent regeneration (24).

**Figure 1:**
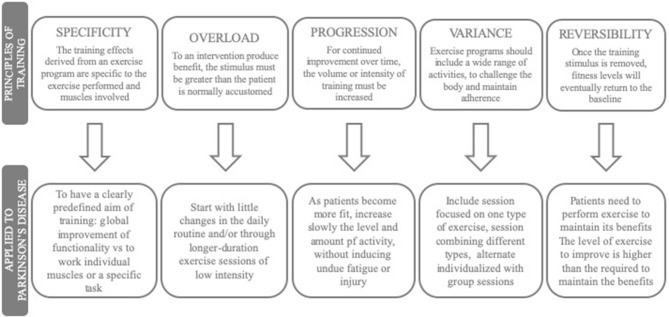
Major aspects of exercise prescription.

To reach it, the major principles of exercise prescription, from sports science field, should be followed. These are specificity, overload, progression, variance, and reversibility (Figure 1) (25, 26).

### Specificity

The principle of specificity states that the effects of training derived from an exercise program are particular to the muscular, cardiorespiratory, and neurologic responses required by the exercise performed, although they may indirectly affect the whole body (22, 26). These should be the first to keep in mind when designing an exercise program and requires to have clear the predefined aim of the training (22, 26).

Most exercise interventions in PD address the major muscle groups and are focused on a more global and functional approach, however, a more focused and individualizes work of some disease-related aspects, is also needed. Patients need to perform functional exercises that improve gait, balance, transfers, and physical capacity. Due to the physical deconditioning associated with a sedentary lifestyle and age-related skeletal muscle atrophy, a more focused work on some specific muscles it's also important (17, 27–29).

### Overload and Progression

The overload and progression principles state that for an intervention to induce improvement, or continue improving, a tissue or organ functional capacity and/or efficiency, the stimulus must be greater than the patient is normally accustomed to in order to induce supercompensation (22, 26).

These two principles are operationalized through the principle of prescription known as the FITT principle (frequency, intensity, type, and time (duration) of training), which brings exercise prescription closer to the management of other therapeutic interventions, i.e., both should follow a detailed and accurate rationale (30).

Due to the lack of standardization of the interventions and low methodological quality of the studies, currently, it is not yet possible to establish what is the most effective way of operationalizing the FITT principle in PD (13, 21, 22). Although with the necessary adjustments, the recommendation from The Centers for Disease Control (CDC) and the American College of Sports Medicine (ACSM), extensible to people with PD, could be used (22, 31). According to these, patients should practice endurance exercise of moderate-intensity for 30–60 min/day on ≥5 days/week for a total of ≥150 min/week, or 20–60 min/day of vigorous-intensity endurance training ≥3 days/week (≥75 min/week). Resistance and flexibility exercises should be performed 2–3 days/week for each of the major joint and muscle groups, and 2–3 days/week of multicomponent exercise involving balance, agility, and coordination (22, 31).

Since the PD patients' levels of PA are usually lower than healthy controls, the stimulus needed to induce overload is usually lower, being frequently reached with little changes in the daily routine and/or through longer exercise sessions of low intensity (20, 21).

### Variance

The principle of variance requires that exercise programs include a wide range of activities so that the body undergoes different challenges and thereby maintains the individual's interest in the program (22).

The FIIT principle has shown to have little or very small influence on patients' adherence to exercise programs (31). On the contrary, individualized, diversified exercise programs according to individual preferences and enjoyment are critical for adopting and adhering to an exercise program (32). Variance could be achieved by performing different types of exercises in different sessions or by combining one or more types in the same session. It could also be reached alternating individualized training with group sessions, or varying exercises, within the same type of training (21, 31).

Evidence on exercise adherence in healthy adults, that may be extrapolated to PD, suggests that: (1) the type of exercise has no or very minimal impact on adherence; (2) adherence is higher in moderate-intensity compared with vigorous-intensity exercise; (3) exercise above the ventilatory threshold have a negative impact on adherence; (4) supervised exercise enhances adherence; (5) exercise environments with attractive distractions, like television, or music, increase adherence; (6) structured home-based programs improve exercise behavior; and (7) strategies to enhance adherence to home- or community-based interventions (e.g., pedometers, telecommunications, group support) show to be effective (31).

### Reversibility

Like any other pharmacological interventions, clinical exercised has a limited duration of action. According to the principle of reversibility, when the training stimulus stops, fitness levels may return to baseline (22, 26). Therefore, PD patients need to continue exercising to maintain the benefits (23).

According to a 2017 review on the long-term effects of exercise in PD: (1) balance training is associated with the longest carry-over effect (up to 12 months after completion of treatment) when compared with the other types of training; (2) the majority of progressive resistive and aerobic training programs produce benefits that last for 12 weeks; and (3) the positive effects of gait training on gait performance and walking capacity remain up to 6 months after training completion (10). Evidence also suggests that is needed a higher level of exercise to obtain cardiorespiratory fitness and cardiometabolic health benefits than to maintain those same benefits (31).

## Are Exercise Programs for PD Patients Safe?

The individual response to physical stimuli can vary significantly depending on intrinsic characteristics and health status, especially in a heterogeneous population, such as PD (22). Therefore any safe and effective exercise prescription needs to take into account the application of training principles, the individual's functional capacity, health status, medication, individual preferences, and goals (31, 33).

The major problems associated with exercise practice are musculoskeletal injuries and cardiovascular risks. To minimize these risks, patients may benefit, especially beginner exercisers, from an individualized evaluation of fitness levels, performed by skilled personnel, with specific instruments, and validated tests (24).

Two recent reviews on physiotherapy and exercise interventions highlighted underreporting of the presence/ absence of adverse events (in both, more than half of the included studies, did not report on adverse events) (23, 34). In our opinion, this may be associated with different attitudes of researchers, regarding safety issues, in non-pharmacological trials compared with drug trials.

## What Is Lacking for Treating PD Patients Like Athletes?

Being the most promising therapeutic intervention for delaying disease progression with good results managing PD symptoms that respond poorly to drug therapy, the confirmation of both symptomatic, and disease-modifying effects of clinical exercise could have a profound societal impact (6, 35). Indeed, one has to bear in mind the good safety profile, easy access, low cost, and the promotion of social interactions associated with clinical exercise interventions (6, 35).

Despite this, the majority of patients are not engaged with the exercise program. Evidence points to four main causes for the underuse of exercise in PD symptom management: (1) the absence of both a clear definition of exercise as a therapeutic intervention and of standardized exercise programs; (2) a lack of recommendations on the best trial design and recommended outcomes to measure exercise efficacy; (3) a lack of knowledge on how to prescribe and implement exercise programs in order to optimize efficacy and adherence; and (4) a lack of awareness and/or recognition by many health professionals and patients of the therapeutic potential of clinical exercise (31, 36).

For patients to begin and maintain daily physical activity and engage in a structured exercise program, they need it to be recommended by health professionals. Since the majority of PD patients are elderly, without exercise habits, it is important to motivate them and ensure compliance, to discuss both the goals and the rationale behind an exercise prescription (20, 22). For those patients more sedentary and resistant to exercise, health professionals may start promoting all possible forms of PA, before recommending a structured exercise program (23, 37).

Exercise development programs have mainly been driven by observational or investigator-initiated trials without clear recommendations on the definition of exercise as a therapeutic intervention, the best trial design, or the recommended outcomes (31, 38). According to an analysis, conducted by our group, of all clinical trials assessing exercise intervention in PD registered in the WHO International Clinical Trials Registry Platform and the ClinicalTrials.gov registries, from 2000 to 2017, almost 70% of studies did not report their findings, and only 25.8% published the results in scientific journals. From the published studies on exercise in PD, 53% of the studies were evaluated as high or uncertain risk of bias in at least half of the domains of the Cochrane Risk of Bias assessment tool (23).

In our view, these limitations represent one of the bottlenecks for further development of exercise in clinical populations. We advocate that all science behind the design and undertaking of pharmacological trials should be brought used to develop exercise programs in order to guarantee the same rigor and precision as required in the development of new medicines. We also believe that the resolution of the underlying gaps in knowledge will require a joint approach from the most relevant scientific and clinical fields, such as movement disorders, sports science fields, and experts in clinical trial methodology.

## Conclusion

Exercise has been hailed as a new therapeutic intervention in PD. However, as with any other therapeutic intervention, its efficacy depends on the way it is prescribed and implemented. To optimize exercise efficacy, a better understanding of the principles of sports science and how to apply them in PD is needed. In order to be consensually accepted as part of the PD armamentarium, exercise development programs should follow the same methodology of other new medicines. For that, we also recommended multidisciplinary collaboration.

## Author Contributions

RB-M conceptualized the paper, drafted manuscript, and revised manuscript. MV, MT, and FS revised manuscript. JF conceptualized the paper and revised manuscript.

### Conflict of Interest

The authors declare that the research was conducted in the absence of any commercial or financial relationships that could be construed as a potential conflict of interest.
